# Dynamic regulation of uncoupling protein 2 expression by microRNA-214 in hepatocellular carcinoma

**DOI:** 10.1042/BSR20160062

**Published:** 2016-05-20

**Authors:** Guangsheng Yu, Jianlu Wang, Kesen Xu, Jiahong Dong

**Affiliations:** * Department of Hepatobiliary Surgery, Shandong Provincial Hospital affiliated to Shandong University, Jinan 250021, China; † Department of Hepatobiliary Surgery, Qilu Hospital of Shandong University, Jinan 250012, China; ‡ Department of Hepatobiliary Surgery, Chinese General PLA Hospital, Beijing 100853, China

**Keywords:** cell, hepatocellular carcinoma (HCC), HepG2, uncoupling proteins, uncoupling protein 2 (UCP2)

## Abstract

Gemcitabine (GEM), a commonly used chemotherapeutic agent in hepatocellular carcinoma (HCC) patients, uses oxidative stress induction as a common effector pathway. However, GEM alone or in combination with oxaliplatin hardly renders any survival benefits to HCC patients. We have recently shown that this is part due to the overexpression of the mitochondrial uncoupling protein 2 (UCP2) that in turn mediates resistance to GEM in HCC patients. However, not much is known about regulatory mechanisms underlying UCP2 overexpression in HCC. Differential protein expression in HCC cell lines did not show a concomitant change in *UCP2* transcript level, indicating post-transcriptional or post-translational regulatory mechanism. *In situ* analysis revealed that *UCP2* is a putative target of *miR-214*. *miR-214* expression is significantly down-regulated in HCC patient samples as compared with normal adjacent tissues and in cell line, human hepatoblastoma cells (HuH6), with high UCP2 protein expression. We demonstrated using *miR-214* mimic and antagomir that the miRNA targeted *UCP2* expression by directly targeting the wild-type, but not a *miR-214* seed mutant, 3’ UTR of *UCP2*. Overexpression of *miR-214* significantly attenuated cell proliferation. Finally, analysis in 20 HCC patients revealed an inverse correlation in expression of *UCP2* and *miR-214* (Pearson's correlation coefficient, *r*=−0.9792). Cumulatively, our data indicate that in the context of HCC, *miR-214* acts as a putative tumour suppressor by targeting *UCP2* and defines a novel mechanism of regulation of *UCP2*.

## Abstract (precis)

Our data indicate that in the context of HCC, *miR-214* acts as a putative tumour suppressor by targeting UCP2 and defines a novel mechanism of regulation of UCP2.

## INTRODUCTION

A widely expressed subcategory of mitochondrial anion-carrier in animals and plants is the uncoupling protein (UCP) family, with the mammalian genome encoding uncoupling protein 1 (UCP1) to uncoupling protein 5 (UCP5) homologues [1–3]. The most ubiquitous among these five homologues is uncoupling protein 2 (UCP2), with detectable expression in skeletal muscle, brain, pancreas, liver and immune cells [4]. *UCP2* is located in chromosome 11q13.4 and encodes for a protein of 309 amino acids and predicted molecular mass of 33.299 kDa. UCP2 is largely expressed in the inner mitochondrial membrane, but expression is also noted in the nucleus, peroxisome, cytosol and plasma membrane [4].

UCP2 in conjunction with uncoupling protein 3 (UCP3) function in suppressing electron transport chain mediated generation of reactive oxygen species (ROS) [5,6]. Physiological levels of ROS are involved in a multitude of cellular functions, inclusive of inflammation, apoptosis, phagocytosis and proliferation [7]. However, overproduction of ROS leads to oxidative damage [8].

Given this intricate role of UCPs in maintaining ROS homoeostasis and cell cycle progression, it is hardly surprising that their aberrant expression have pro-tumorigenic effects on the cell [9]. UCP2 is found to be overexpressed in hepatocellular carcinoma (HCC) [10] and colon cancer [11]. In colon cancer cells, UCP5 is also overexpressed [12]. Current evidence suggests that UCP2 targets p53 and reverses pro-apoptotic signals initiated by p53 in response to oxidative stress [13]. We have recently shown that UCP2 expression mediates resistance to Gemcitabine (2’,2’-difluoro-2’-deoxycytidine; GEM), which is used in combination with oxaliplatin as chemotherapeutic agents in HCC and that inhibition of UCP2 makes HCC cell lines susceptible to treatment with GEM [14].

Given the important role of UCP2 in HCC, it is imperative to understand the regulatory mechanisms that dictate expression of UCP2 in HCC. Our experiments have cumulatively shown that UCP2 transcript is post-transcriptionally regulated by *miR-214* in normal hepatic cells and that down-regulation of *miR-214* in HCC induces UCP2 expression in these HCC cells.

## MATERIALS AND METHODS

### Clinical samples, tissue processing and ethical considerations

Fresh-frozen and paraffin-embedded HCC tissues and corresponding adjacent non-tumorous HCC tissue samples were obtained from 25 Chinese patients at Qilu Hospital of Shandong University between 2010 and 2014. All cases were included post review by pathologist and histological confirmation as HCC and only where complete clinical pathology and follow-up data were available. None of the 25 included patients underwent preoperative local or systemic treatment. The study protocol was approved by the Institutional Review Board of the Qilu Hospital of Shandong University. Freshly harvested samples were immersed in RNAlater (Life Technologies) before snap freezing within 30 min post-surgery. All tissue samples were stored in liquid nitrogen until further use.

### Cell culture

HCC cell lines human hepatoblastoma cells (HuH6) and human lens epithelial cells (HLE) were obtained from the A.T.C.C. and maintained at 37°C in a CO_2_ incubator in Dulbecco's modified Eagle's media (DMEM) supplemented with 10% FBS (Gibco) and 100 I.U./ml penicillin and 100 μg/ml streptomycin (Gibco).

### Isolation of mitochondria

Isolation of mitochondria from different cell lines was as recently and previously described [14,15].

### RNA and miRNA extraction and quantitative real-time PCR

Total RNA was isolated from cultured cells and tumour tissues using Trizol reagent. First strand cDNA was synthesized using the RevertAid™ First Strand cDNA synthesis Kit (Life Technologies), which was then used for real-time PCR using TaqMan Gene Expression probes (Life Technologies). 18s rRNA (TaqMan Assay ID: Hs03003631_g1) was used as an internal control for assessing UCP2 (TaqMan Assay ID: Hs01075227_m1) transcript level. Data were normalized to 18s rRNA expression and analysed by the −ΔΔ*C*_t_ method. According to the manufacturer's instructions, miRNA from tissues and cells was extracted using the mirVana miRNA isolation kit (Life Technologies) and the expression levels of *hsa-miR-214* and U6 small nuclear RNA (RNU6B) were detected by TaqMan miRNA assays (Life Technologies) (TaqMan Assay IDs: 002306 and 001093 respectively). Data were normalized to RNU6B expression and analysed by the −ΔΔ*C*_t_ method.

### Determination of mRNA stability

HuH6 and HLE cells were treated with 10 μM Actinomycin-D (Sigma–Aldrich) for 0.5, 2, 4, 6, 8, 10 or 12 h before RNA isolation. Amount of *UCP2* levels in the isolated mRNA samples were determined by quantitative real-time PCR as described above and compared with levels in untreated samples from the same cells. Relative expression was normalized to *TBP* (TaqMan Assay ID: Hs00427620_m1) in the same samples and data were converted into percent mRNA left at the indicated time points.

### Western blot analysis

Western blot analysis was performed as described previously using rabbit anti-UCP2 antibody (Santa Cruz Biotechnology) [16,17]. All membranes were probed with anti-GAPDH (glyceraldehyde-3-phosphate dehydrogenase) antibody (Santa Cruz Biotechnology) to confirm equal protein loading.

### Cell proliferation assays

Cell proliferation was quantified using a mitochondrial colorimetric assay (MTT assay, Sigma–Aldrich) as per the manufacturer's recommendations and as described recently [14]. Results from three independent triplicates were expressed as mean ± S.D.

### Plasmids

The UCP2 3' UTR clone in pMirTarget was obtained from Origene. The UCP2 3' UTR reporter was constructed by amplifying the endogenous UCP2 3' UTR from the Origene clone. XhoI and ApaI sites were added to the 5'- and 3'- ends of the fragment during the preceding PCR reaction and cloned into the XhoI and ApaI site on the Rr-luc-6XCXCR4 (Addgene plasmid 11308) Renilla luciferase vector. To make the UCP2 3’ UTR mutant construct, site-directed mutagenesis was used to delete 6–16 region, corresponding to the *hsa-miR-214*-binding site. A firefly luciferase vector was used as transfection and normalization control in all luciferase assays. Constructs were sequence verified to University of California Santa Cruz (UCSC) human genome reference version human genome (hg19).

### Transfection and luciferase assays

Cells (4×10^4^) were transiently transfected with the luciferase reporter constructs using Lipofectamine LTX (Life Technologies) as per the manufacturer's instructions. Where indicated, cells were transfected with the *miR-214* mimic or antagomir (Life Technologies) along with the UCP2 3’ UTR constructs. Forty-eight hours after transfection, the renilla and firefly luciferase activities were measured consecutively using Dual-luciferase reporter assay system (Promega) as per manufacturer's protocol. Each reporter plasmid was transfected at least twice (on different days) in triplicate. Post-normalization, the data were represented as relative fluorescence units (RFU) ± S.D.

### Statistical analyses

SPSS version 20.0 (IBM) was used for all statistical analysis. Two-sided *P*-values <0.05 were considered statistically significant.

## RESULTS

### UCP2 transcript is targeted by *miR-214*

**Figure 1:**
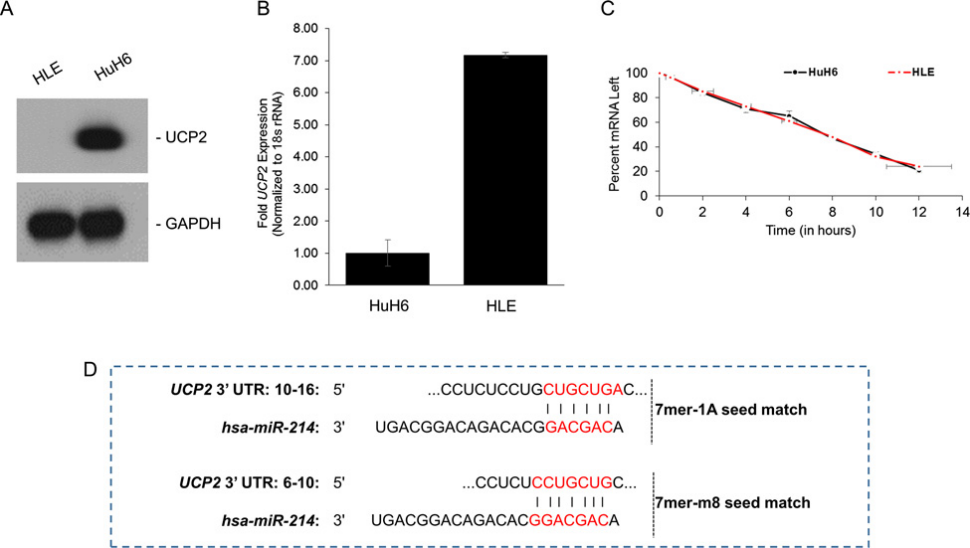
UCP2 expression is post-transcriptionally regulated in liver cancer cells (**A**) Basal expression levels of UCP2 in mitochondrial extracts obtained from indicated HCC cell lines. The blot was stripped and probed with GAPDH to serve as a loading control. (**B**) Steady state expression of *UCP2* mRNA was determined in indicated cell lines. Data were normalized to 18s rRNA expression. (**C**) HuH6 and HLE cells were treated with Actinomycin-D for indicated times to determine relative stability of *UCP2* transcript in the two cell lines. The slope of the two cell lines showed that degradation of the *UCP2* mRNA in either cell lines followed similar kinetics. (**D**) Complementary 7mer-1A and 7mer-m8 seed match between *miR-214* and the 3’ UTR of *UCP2* as predicted by TargetScan software.

We have recently observed differential UCP2 protein expression among different HCC cell lines [14]. Whereas robust steady state UCP2 protein expression was detected in HuH6 cells, it was suppressed in the HLE cells (Figure 1A). Assessment of UCP2 transcript levels indicated that the difference in protein expression was not due to differential transcription rates. In fact, UCP2 transcript was significantly overexpressed (7±0.3-fold, *P*<0.05) in HLE cells as compared with the HuH6 cells (Figure 1B). This indicated a post-transcriptional or post-translational regulatory mechanism underlying differential UCP2 protein expression in these cells.

Evaluation of mRNA stability following Actinomycin-D treatment did not reveal any significant difference in UCP2 half-life in the two cell lines (Figure 1C). We next wanted to determine if UCP2 is being targeted by miRNAs. *In situ* prediction using TargetScan platform [18] showed that *miR-214* have two putative and adjacent binding sites in the 3’ UTR of UCP2 (Figure 1D).

### *miR-214* is down-regulated in HCC samples

**Figure 2:**
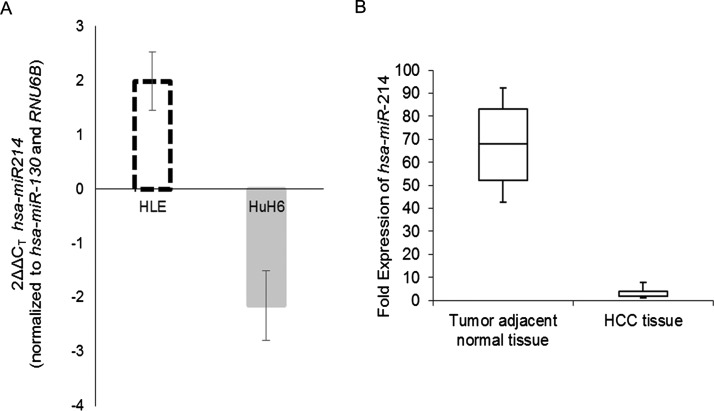
*miR-214* expression is down-regulated in HCC Steady state expression of *miR-214* in indicated cell lines (**A**) or paired tumour and adjacent non-tumour tissue (**B**) were determined. Data were normalized to *RNU6B* expression.

Quantitative real-time PCR showed that *miR-214* expression was up-regulated in HLE cells and suppressed in HuH6 cells (Figure 2A). Evaluation of *miR-214* expression in 25 paired HCC and adjacent normal tissue specimens showed that *miR-214* expression was significantly down-regulated in HCC tissue (median, 6.39; range, 1.25–9.01) compared with normal counterparts (median, 68.87; range, 38.17–91.42) (*P*<0.001) (Figure 2B).

### Modulating *miR-214* levels impacted proliferation in the HCC cells

**Figure 3:**
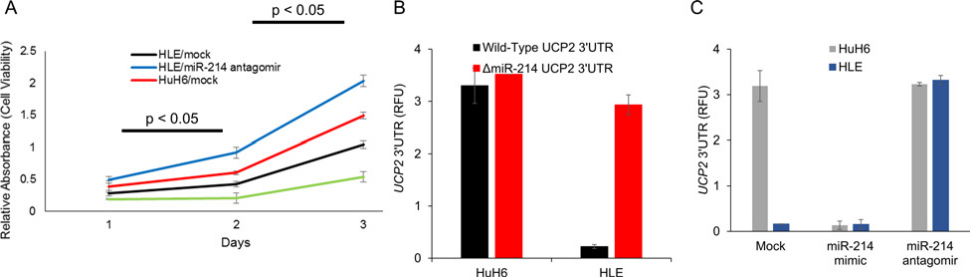
*UCP2* is a bona fide target of *miR-214*, expression level of which control cell viability (**A**) Cell viability was measured in HLE and HuH6 cells at 24, 48 and 72 h after transfection with *miR-214* antagomir or mimic respectively, by the MTT assay. (**B**) Relative luciferase activity of transiently transfected luciferase reporter constructs containing either full-length or mutated (*miR-214*-binding sites deleted) *UCP2* 3’ UTR in indicated cells. (**C**) Relative luciferase activity of transiently transfected luciferase reporter constructs containing full-length *UCP2* 3’ UTR in indicated cells, alone or in combination with *miR-214* mimic and antagomir.

Since UCP2 can inhibit ROS-induced apoptosis [19], we rationalized that altering UCP2 transcript levels by modulating *miR-214* expression might affect proliferation rates. This led us to examine whether overexpression via transfection of *miR-214* mimic in HuH6 cells and suppression via transfection of *miR-214* antagomir in HLE cells would impact proliferation rates. *miR-214* mimic significantly decreased cell viability of HuH6 cells at 24, 48 and 72 h post-transfection respectively, compared with the mock control (*P*<0.05 in each case). Vice versa, *miR-214* antagomir induced significantly more cell proliferation in HLE cells at the indicated time points (*P*<0.05 in each case) (Figure 3A).

### UCP2 is a direct target of *miR-214* in HCC cells

We next determined if UCP2 is a bona fide target of *miR-214* in HCC cell lines. To test this putative interaction, luciferase reporter constructs containing the wild-type UCP2 3’ UTR were transfected in HuH6 and HLE cells (Figure 3B). UCP2 3’ UTR containing reporter were inhibited 9.8±0.34-fold (*P*=0.0037) in HLE cells compared with the HuH6 cell line. To confirm that the effects observed was due to *miR-214* targeting the UCP2 3’ UTR, we generated and tested a *miR-214* binding mutant of the UCP2 3’ UTR reporter, in which both the putative binding sites between 6–16 nucleotides were deleted. The *miR-214* binding mutant UCP2 3’ UTR reporter did not show any difference in relative luciferase activity between HLE and Huh cells (Figure 3B), confirming that UCP2 mRNA was being targeted by the *miR-214* in these cells. This was further corroborated by reporter assays performed in HLE cells transfected with *miR-214* mimic and HuH6 cells transfected with *miR-214* antagomir. Whereas *miR-214* mimic inhibited UCP2 3’ UTR reporter (*P*<0.05) in HuH6 cells, *miR-214* antagomir rescued reporter activity in the HLE cells (*P*<0.05) (Figure 3C).

### *miR-214* expression is inversely correlated with UCP2 levels and HCC disease

**Figure 4:**
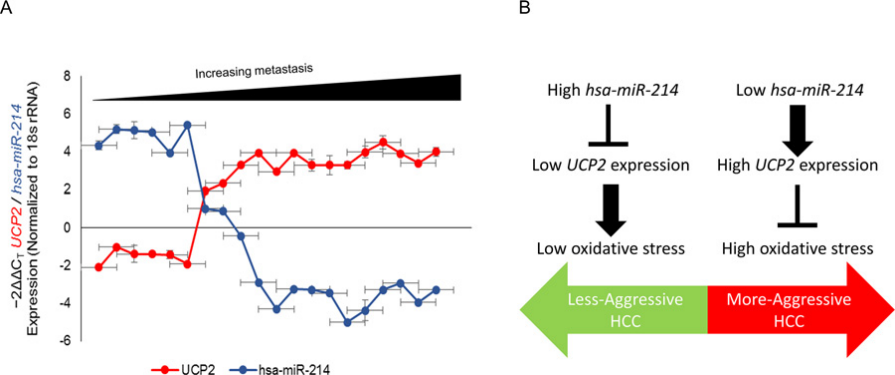
*UCP2* mRNA and *miR-214* are inversely correlated in patients with HCC (**A**) Pearson correlation demonstrating the inverse relation between *UCP2* and *miR-214* in paired samples (*P*<0.005, Pearson correlation, *r*=−0.9792). (**B**) Model illustrating the relationship between expression level of *miR-214* and *UCP2*, mitochondrial superoxide generation and HCC.

Given that our experiments indicated that UCP2 is a bona fide target of *miR-214*, we hypothesized that suppression of *miR-214* expression might be an underlying feature of human prostate cancer. We determined *miR-214* and UCP2 expression in 20 HCC patients, ten with high *miR-214* and ten with low *miR-214* expression. The ones with high *miR-214* expression corresponded to N0, N1 cases (non-metastatic) whereas those with low *miR-214* expression corroborated to highly metastatic disease. Our results indicated a dynamic and inverse correlation between down-regulation in the levels of *miR-214* and the observed increase in the expression of UCP2 in HCC tissue specimens (Figure 4A) (*P*<0.005, Pearson correlation, *r*=−0.9792).

## DISCUSSION

miRNAs are evolutionarily conserved 21–23 nucleotides RNAs that regulate post-transcriptional gene expression either by blocking translation or degrading target mRNAs and have been increasingly shown to function as tumour suppressors or oncogenes [20,21]. miRNAs can function in both normal and transformed cells and have even been shown to play a role in metastasis [22–25].

Regulation of factors participating in ROS homoeostasis by miRNA is not without prior precedence. It has been shown that during progression from adaptive hypertrophy to heart failure, *miR-214* and miR-30* together regulate cardiac vascular endothelial growth factor (VEGF) expression and angiogenesis by targeting X-box-binding protein-1 (XBP1), a key transcription factor of the unfolded protein response in mammalian cells [26].

Our results suggest that in the context of HCC, *miR-214* functions as a tumour suppressor (Figure 4B). However, along with *miR-126*, *miR-214* has been shown to be overexpressed in malignant endothelial proliferative disease [27]. This presents a unique case where the same miRNA can function as a tumour suppressor or oncomir in a context-dependent fashion. Elucidating the underlying mechanism regulating *miR-214* expression will help explain the differential functional readouts of *miR-214* in malignant proliferative disease and HCC.

Our prediction of *miR-214* targeting *UCP2* mRNA was based on the TargetScan algorithm. However, according to the miRTarBase, miRNAs that target *UCP2* are *hsa-miR-15a-5p* and *hsa-miR-484*. Similarly, according to the miRanda algorithm *miR-497*, *miR-15a*, *miR-424*, *miR-195*, *miR-16* and *miR-15b* can target *UCP2*, accessed on February 25, 2016. Experiments have shown that five programmes, namely TargetScan, TargetScanS, PicTar, DIANA-microT and miRNA target genes database (EIMMO) had a specificity of approximately 50% and sensitivity ranging from 6–12% [28]. Our prediction of *miR-214* was through one of these five algorithms. However, it is important for future studies to validate if other miRNAs target *UCP2* mRNA in the context of HCC or otherwise.

UCP2 is known to suppress ROS level which is overexpressed by various types of cancer cells including HCC cell lines. Inhibition of UCP2 in cancer cells have been shown to increase susceptibility of drug-resistant cancer cells to cytotoxic agents [19,29], indicating UCP2 is overexpressed in these cancers. It will be important to confirm if *miR-214* levels are also down-regulated in these cells or UCP2 expression is controlled by additional mechanism.

## Abbreviations

GAPDH: glyceraldehyde-3-phosphate dehydrogenase
GEM: Gemcitabine
HCC: hepatocellular carcinoma
HLE: human lens epithelial cells
HuH6: human hepatoblastoma cells
MTT: mitochondrial colorimetric assay
RNU6B: U6 small nuclear RNA
ROS: reactive oxygen species
UCP2: uncoupling protein 2
UCP5: uncoupling protein 5
